# Vaccination of Lactating Dairy Cows for the Prevention of Aflatoxin B_1_ Carry Over in Milk

**DOI:** 10.1371/journal.pone.0026777

**Published:** 2011-10-28

**Authors:** Luciano Polonelli, Laura Giovati, Walter Magliani, Stefania Conti, Stefano Sforza, Alessandro Calabretta, Claudio Casoli, Paola Ronzi, Ester Grilli, Antonio Gallo, Francesco Masoero, Gianfranco Piva

**Affiliations:** 1 Sezione di Microbiologia, Dipartimento di Patologia e Medicina di Laboratorio, Università degli Studi, Parma, Italy; 2 Dipartimento di Chimica Organica ed Industriale, Università degli Studi, Parma, Italy; 3 Dipartimento di Scienze Cliniche L. Sacco, Sezione di Malattie Infettive e di Immunopatologia, Università degli Studi, Milano, Italy; 4 Dipartimento di Scienze Mediche Veterinarie, Università degli Studi di Bologna, Bologna, Italy; 5 Istituto di Scienze degli Alimenti e della Nutrizione, Facoltà di Agraria, Università Cattolica del Sacro Cuore, Piacenza, Italy; Oxford University, United Kingdom

## Abstract

The potential of anaflatoxin B_1_ (AnAFB_1_) conjugated to keyhole limpet hemocyanin (KLH) as a vaccine (AnAFB_1_-KLH) in controlling the carry over of the aflatoxin B_1_ (AFB_1_) metabolite aflatoxin M_1_ (AFM_1_) in cow milk is reported. AFB_1_ is the most carcinogenic compound in food and foodstuffs amongst aflatoxins (AFs). AnAFB_1_ is AFB_1_ chemically modified as AFB_1_-1(O-carboxymethyl) oxime. In comparison to AFB_1_, AnAFB_1_ has proven to be non-toxic in vitro to human hepatocarcinoma cells and non mutagenic to *Salmonella typhimurium* strains. AnAFB_1_-KLH was used for immunization of cows proving to induce a long lasting titer of anti-AFB_1_ IgG antibodies (Abs) which were cross reactive with AFB_1_, AFG_1_, and AFG_2_. The elicited anti-AFB_1_ Abs were able to hinder the secretion of AFM_1_ into the milk of cows continuously fed with AFB_1_. Vaccination of lactating animals with conjugated AnAFB_1_ may represent a solution to the public hazard constituted by milk and cheese contaminated with AFs.

## Introduction

Mycotoxins are secondary metabolites produced by molds, classified among the most important risk factors in the food chain of humans and animals. The problem of mycotoxicoses is global and is particularly affecting the countries characterized by environmental and weather conditions favourable to contamination and growth of fungi both in field and storage of stocks. It has been estimated that 25% of the world's food crops is contaminated with mycotoxins, and more than 4.5 billion people and an undefined number of animals are chronically exposed to aflatoxins (AFs), the most relevant mycotoxins of medical interest [1], [2]. AFs (AFB_1_, AFB_2_, AFG_1_, AFG_2_), produced mainly by strains of *Aspergillus flavus* and *A. parasiticus*, may cause acute (hepatitis, oedema, hemorrhagic necrosis) or chronic (liver, lung, and kidney carcinomas and immunosuppression) toxic effects [2]. The main AF, AFB_1_, has a range of biological activities, including acute toxicity, teratogenicity, mutagenicity and carcinogenicity [3]. The International Agency for Research on Cancer (IARC) has classified AFB_1_ as the most important known carcinogenic compound (group 1), particularly related to hepatocarcinoma [4], [5].

In animal farming, AFs may cause reduced performance, increased susceptibility to infections, altered responsiveness to vaccinations [6], in addition to contamination of derived dietary products such as meat, eggs and milk [7], [8]. Following ingestion of contaminated feed, AFB_1_ is rapidly adsorbed and transported to the liver where it is partially metabolized into the hydroxy-derivate M_1_, which may be secreted in the milk of mammals, including dairy animals (carry over process) [9], [10]. AFM_1_, which is as toxic as AFB_1_, has been included in Group 2 (potentially carcinogenic for humans) by IARC [4]. By association with casein, AFM_1_ occurring in the milk concentrates during cheese making, thus increasing the risk potential of the milk/cheese production chain [11].

Industrialized countries have defined specific limits for AFM_1_ in the milk destined for human consumption (ranging from 0.05 µg AFM_1_/kg of the European Community to 0.5 µg/kg of USA), and for AFB_1_ in dairy animal feeds [12]. The best strategy to counter the AFs problem is the prevention of fungal contamination in the food chain. When outbreaks of AFs occur, some means of salvaging contaminated feeds involve physical or chemical detoxifying methods or inclusion in the diet of a variety of animals of sequestering agents, able to prevent AFs absorption in the gastro-intestinal tract [13]. However, none of these methods fulfill completely the efficacy, safety, and cost requisites of the task [14]. The production of a vaccine able to induce specific antibodies (Abs) neutralizing AFB_1_ and other AFs would be of great social, scientific and economic interest. Conventional vaccine approaches are not feasible due to the non immunogenicity of AFB_1_. Its conjugation to proteins, shown to be effective with other haptens, would be unlikely for human use and hardly proposable in animals, owing to the toxic properties of the molecule that might be released. A modified form of AFB_1_ (anaflatoxin B_1_, AnAFB_1_), devoid of toxicity and mutagenicity, still maintaining antigenicity when conjugated, would be a potential vaccine candidate. A method for the preparation and purification of AFB_1_-1(O-carboxymethyl) oxime from AFB_1_ has been described, and the derivative was shown to be nontoxic to chicken embryos [15]. Lower mortality and reduction of acute toxic effects in the liver was achieved in rabbits and rats immunized with AFB_1_-1(O-carboxymethyl) oxime conjugated to bovine serum albumin (BSA) or histone H1 and challenged with a single dose of AFB_1_ [16], [17], [18].

The aim of this study was to evaluate whether AnAFB_1_, represented by AFB_1_-1(O-carboxymethyl) oxime, verified *in vitro* to be nontoxic to human hepatocarcinoma cells and non mutagenic to *Salmonella typhimurium* strains, might constitute, following conjugation to a non-bovine protein (KLH), a potential vaccine to prevent the carry over of AFB_1_ as AFM_1_ in the milk of dairy cows receiving an AFB_1_-contaminated diet.

## Results

### Cytotoxicity assay of AFB_1_ and AnAFB_1_ on HepG2 cells

**Figure 1:**
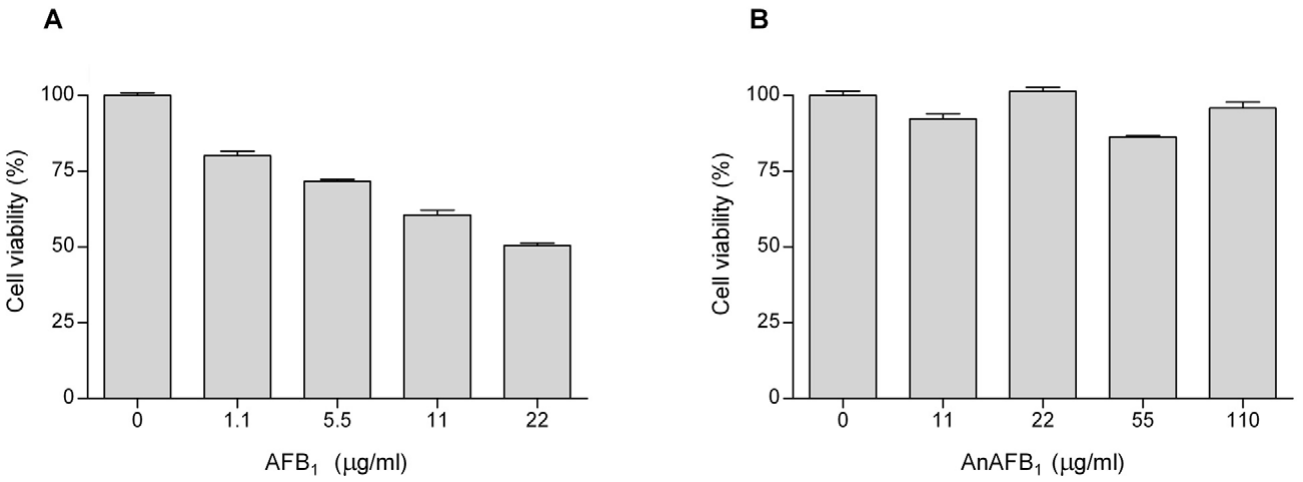
In vitro effects of AFB_1_ (A) and AnAFB_1_ (B) on human hepatoblastoma cell viability.

The effects of a range of concentrations of AFB_1_ and AnAFB_1_ on the viability of HepG2 hepatoblastoma cells were evaluated by a colorimetric assay. AFB_1_ caused a dose-dependent cytotoxicity, as survival of the cells inversely decreased with increases in the concentration of AFB_1_ from 1.1 to 22 µg/ml (Figure 1A). Cells exposed to AnAFB_1_ showed no significant decrease in cell viability at concentrations up to 110 µg/ml (Figure 1B).

### In vitro mutagenicity assay of AFB_1_ and AnAFB_1_ in *Salmonella typhimurium*

The Ames test with strains TA 98 and TA 100 was carried out in order to elucidate whether AnAFB_1_ retained the mutagenic properties of AFB_1_. The results of the mutagenicity assay are shown in Table 1.

**Table 1:** Mutagenicity of AFB_1_ and AnAFB_1_ in *S. typhimurium* TA98 and TA100.

	dose	*his* ^+^ revertants*	
	(ng/plate)	TA 98	TA 100
**AFB_1_**	0	6±2	25±22
	100	175±31	133±21
	200	307±49	622±47
**AnAFB_1_**	0	6±2	25±22
	100	2±1	26±14
	200	2±2	5±2
	500	4±2	59±25

*mean of two independent, triplicate mutagenicity assays with SD.

AnAFB_1_, up to a concentration of 200 ng/plate, did not elicit a mutagenic response either in *S. typhimurium* TA98 or TA 100. At the concentration of 500 ng/plate, a positive, but very weak, mutagenic activity was observed only in *S. typhimurium* TA 100, while the positive control AFB_1_ induced a strong mutagenic response at concentrations of 100 and 200 ng/plate.

### ELISA titration of anti-AFB_1_ Abs

**Figure 2:**
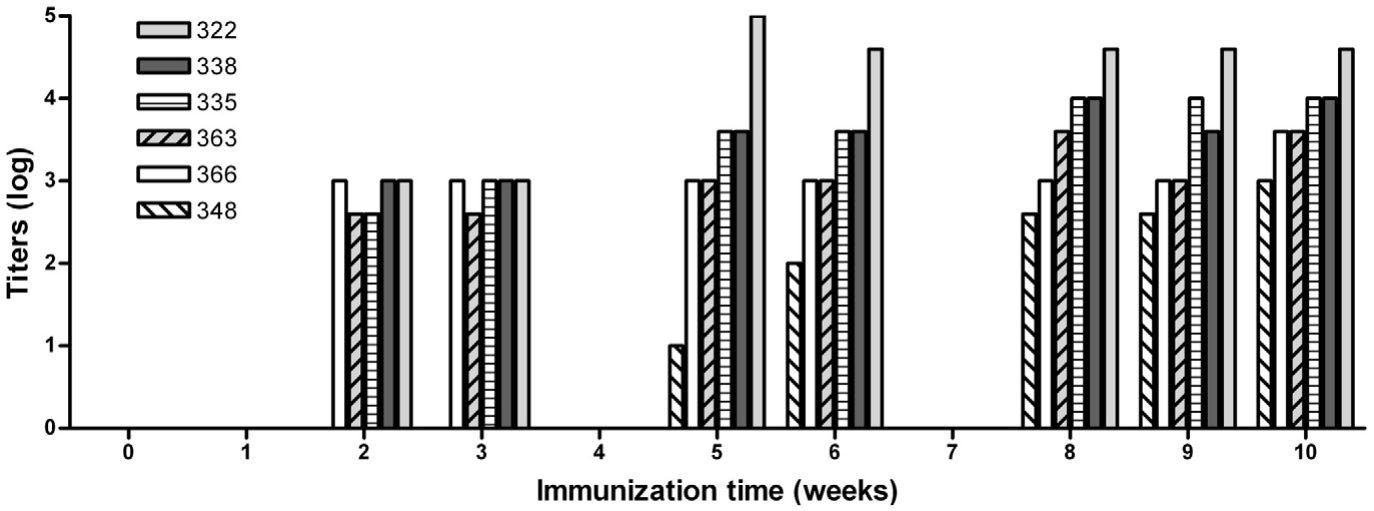
Titers of anti-AFB_1_ Abs. Cows numbered 322, 335, 338, 348, 363 and 366 were initially *i.m.* primed with 500 µg of AnAFB_1_-KLH conjugate and then boosted at week 3, 6 and 9 with the same amount of immunogen. Ab titers (presented in figure on a logarithmic scale) were determined by the method described in the text.

Production of Abs against AFB_1_ in vaccinated and control dairy cows was evaluated by ELISA. For all of the cows, dilutions of pre-immune control sera showed negligible binding to AFB_1_-BSA. When dilution series of cow sera were incubated with control BSA (unconjugated), only a low degree of nonspecific binding was detected (data not shown). Control cows (6 out of 6) did not produce, as expected, anti-AFB_1_ Abs (data not shown). Ab titers of vaccinated cows over a 10-week period are presented in Figure 2. Based on the anti-AFB_1_ Ab titer, at the 10^th^ week it was possible to differentiate two groups among vaccinated cows. Animals producing the highest serum titers ranging from 10,000 to 40,000 were defined as “high responders” (cow number 322, 335, 338). The animals presenting titers ranging from 1,000 to 4,000 were defined as “low responders” (cow number 348, 363, 366). Following immunization none of the animals provided positive reactions to the intradermal tuberculin test as attested by the local competent authority.

### Cross-reactivity of anti-AFB_1_ Abs with other AFs

Sera from the 10^th^ week bleeding were selected for cross-reactivity evaluation with AFs. The cross-reactivity of immune sera collected from each cow with AFB_2_, AFG_1_, and AFG_2_ was similar and averaged 17%, 31%, and 9%, respectively (Table 2). AFM_1_ displayed negligible cross-reactivity for all the immune sera, since 50% inhibition of binding to AFB_1_-BSA was not reached using concentrations up to 1000 ng/ml.

**Table 2:** Cross-reactivity of immune sera with AFs.

	Cow number												
	322		335		338		348		363		366		
AF	IC_50_ *	%	IC_50_	%	IC_50_	%	IC_50_	%	IC_50_	%	IC_50_	%	% average
AFB_1_	76.69	100	28.06	100	150.80	100	58.96	100	66.11	100	48.41	100	100
AFB_2_	496.10	15	213.50	13	990.00	15	301.50	20	282.40	23	393.30	12	17
AFG_1_	339.60	23	126.70	22	502.90	30	253.10	23	167.40	39	100.70	48	31
AFG_2_	906.70	8	480.70	6	848.70	18	2611.00	2	730.50	9	405.70	12	9

*IC_50_ is the AF concentration (ng/ml) causing 50% inhibition of binding of the immune serum to the solid phase.

### Quantification of AFM_1_ in milk samples

The efficacy of anti-AFB_1_ Abs in preventing or reducing the carry over of AFB_1_ as AFM_1_ into milk was evaluated by monitoring AFM_1_ concentrations in milk of the lactating dairy cows. An intermittent exposure regimen with two intoxication periods was designed to evaluate efficacy of anti-AFB_1_ Abs over time and in two different lactation (mid and late) stages.

**Figure 3:**
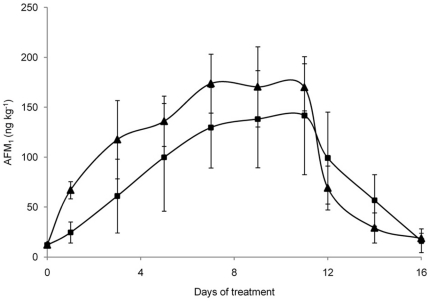
Average AFM_1_ concentration in milk collected during the first experimental period. Six vaccinated (▪) and six control (▴) cows were fed 144 µg of AFB_1_/day from day 1 to day 11. Data are presented as mean ± SD.

**Figure 4:**
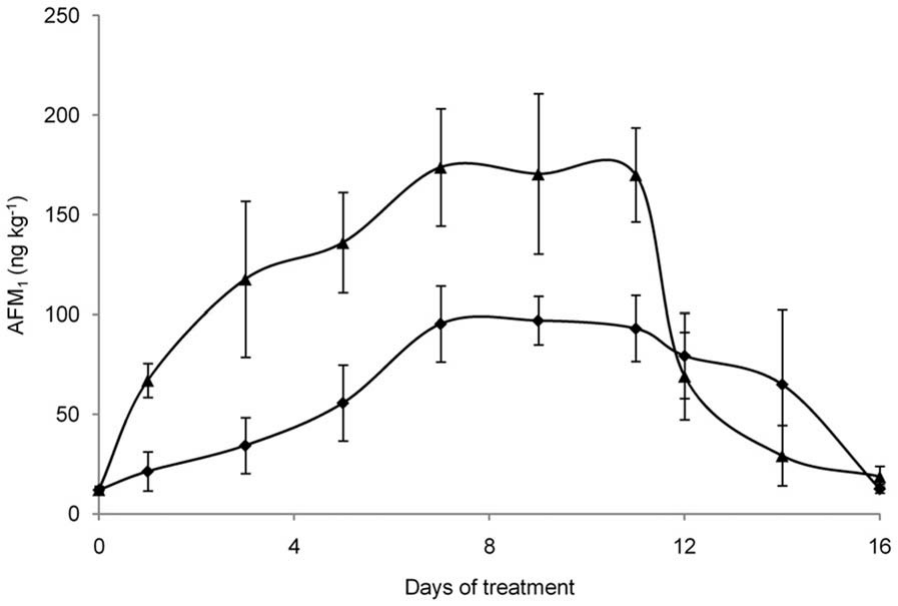
Average AFM_1_ concentrations in milk collected during the first experimental period from high responder cows. Three high responder vaccinated cows (♦) and six control cows (▴) were fed 144 µg of AFB_1_/day from day 1 to day 11. Data are presented as mean ± SD.

**Figure 5:**
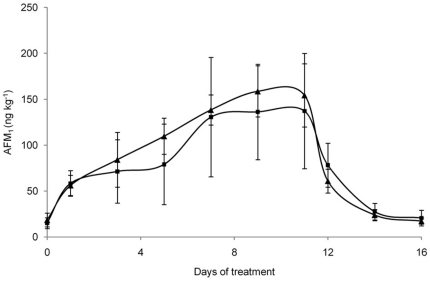
Average AFM_1_ concentrations in milk collected during the second experimental period. Six vaccinated (▪) and six control (▴) cows were fed 145 µg of AFB_1_/day from day 1 to day 11. Data are presented as mean ± SD.

**Figure 6:**
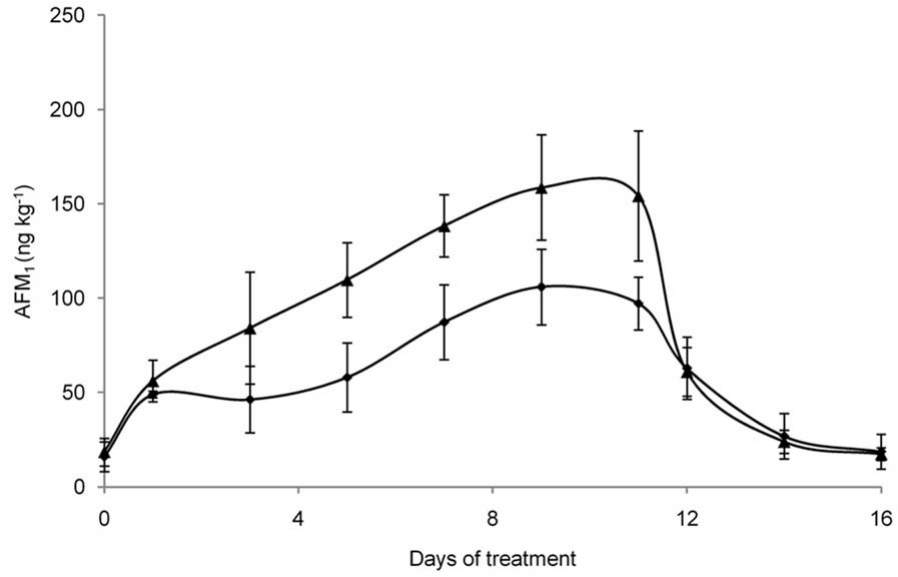
Average AFM_1_ concentrations in milk collected during the second experimental period from high responder cows. Three high responder vaccinated cows (♦) and six control cows (▴) were fed 145 µg of AFB_1_/day from day 1 to day 11. Data are presented as mean ± SD.

Basal diet AFB_1_ level contributed to a milk AFM_1_ contamination of 12.1±1.3 and 16.8±6.6 ng/kg in the first and second period, respectively (as calculated on day 0). Results of AFM_1_ quantification in the milk collected during the first intoxication period (144 µg AFB_1_ *per* cow *per* day) are shown in Figure 3. At day 1, the milk sampled from the control cows had an AFM_1_ concentration higher than the tolerable level allowed by the EC (0.05 µg/kg) [19]. On the contrary, the milk samples collected from vaccinated cows had an AFM_1_ concentration lower than control milk and below the EC maximum allowed level. However, the AFM_1_ concentration in milk increased at every milking and reached a steady-state condition from day 7 of intoxication period for both groups. On day 11, when AFB_1_ administration was stopped, the mean AFM_1_ concentration decreased quickly to return at the base line on day 16. During the experimental period, the milk of vaccinated cows consuming contaminated feed showed AFM_1_ levels always lower (even non statistically significant) than the milk of control animals. In particular, at the steady state condition, the average AFM_1_ concentration in milk collected from vaccinated cows was 20% lower than in milk of control animals (137 ng/kg *vs* 171 ng/kg). A negative correlation between serum AFB_1_ specific Ab titers and the average concentration of AFM_1_ detected in the milk at the steady state condition during the first experimental period (r = −0.72, p<0.05) was observed in vaccinated cows. Importantly, the “high responder” cows, showing the highest titers of anti-AFB_1_ Abs, produced an average milk AFM_1_ concentration at the steady state about 46% lower (p<0.05) than control cows (95 ng/kg *vs* 177 ng/kg; Figure 4). During the second experimental period, AFM_1_ in milk from vaccinated cows consuming the contaminated feed (145 µg AFB_1_ *per* cow *per* day) showed the same trend as in first experimental period, with AFM_1_ levels always lower (even non statistically significant) than that of control cows consuming AFB_1_. In particular, at the steady state condition, average AFM_1_ concentration in milk collected from vaccinated cows was 11% lower than in milk of control animals (134 *vs* 154 ng/kg, respectively) (Figure 5). Similarly to first exposure period, the average concentration of AFM_1_ in milk at the steady state condition was correlated (r = −0.68, p<0.05) with anti-AFB_1_ Ab titers in the serum of vaccinated cows, with average AFM_1_ concentration in milk of “high responder” cows 37% lower (p<0.05) of that observed in control cows (97 *vs* 154 ng/kg, respectively; Figure 6).

## Discussion

The occurrence of AFM_1_ in milk and its derivatives is a serious problem of food safety, as milk is a primary source of human nutrition, in particular for infants and children. To reduce human and animal exposure, industrialized countries have defined specific limits for AFM_1_ in the milk and for AFB_1_ in the feed of dairy animals [12]. Currently, the best strategy in order to avoid exceeding of the maximum limit of AFM_1_ in the milk destined for human consumption is the prevention of AF contamination of feeds, but in spite of the control measures taken, production of AFM_1_-free milk is not always achieved. In industrialized countries the milk having higher levels of contamination than the current legal limits is destroyed, although exposure to low doses of AFM_1_, which could be present beneath that limit, is not prevented, with potential health effects due to accumulation. Moreover, in developing countries, where food availability has often to be considered before food safety, there is a lack of legislation of acceptable limits for AFs and populations are undoubtedly exposed to high amounts of AFM_1_ in milk.

An alternative management of the problem, therefore, could rely on a preventive approach such as a safe and reliable vaccine for dairy cattle which should be effective in avoiding AFB_1_ carry over in the milk.

We report here the proof-of-concept that AnAFB_1_, composed of AFB_1_-1(O-carboxymethyl) oxime, which proved to be non toxic and non mutagenic in controlled experimental conditions, may fulfill with the purpose. AnAFB_1_ conjugated to a carrier protein (KLH) and with Freund's adjuvant elicited in cows cross-reactive anti-AFs long lasting Ig, mostly pertaining to IgG, although other Ig classes could contribute to observed results, since the Abs used to detect anti-AFB_1_ Abs were not gamma chain specific. Anti-AFs Abs proved to substantially reduce AFB_1_ carry over as AFM_1_ in the milk for a prolonged period of ingestion of contaminated feed. No adverse effect on animal health was observed in AFB_1_ exposed cows, consistently with previous works adopting similar contamination levels [20], [21]. It is notable that a single administration of the vaccine in complete Freund's adjuvant did not induce delayed hypersensitivity in any of the vaccinated cows, as demonstrated by negative intradermal tuberculin test. This finding should exclude any misleading evaluation of livestock health.

According to Ab titer specific for AFB_1_, it was possible to recognize, among the 6 vaccinated cows, 3 high responder and 3 low responder animals. The titer of Abs specific to AFB_1_ in vaccinated cows correlated well with the prevention of carry over in the milk, following exposure of the cows to feed contaminated with AFB_1_. Cows of the high responder group (titers ranging from 10,000 to 40,000) presented, at the steady state, significantly lower concentrations of AFM_1_ in the milk than cows of the low responder group (titers ranging from 1,000 to 4,000). Moreover, the anti-AFB_1_ Abs elicited in the high responder cows appeared to reduce the excretion of AFM_1_ in the milk following intoxication either in the mid or late lactation stages, thus conferring protection over the whole production cycle, before the drying off period.

The reasons why healthy, immunocompetent animals may be differently susceptible to immunization are not well understood, but it has been reported that cows could be phenotypically classified as low or high responders, based on the magnitude and kinetics of the Ab response to injection of various antigens [22], [23], [24]. Recognized factors of variation in cows' responsiveness to immunization include, among others, the energy balance derived from feeding regimen, peripartum stress and lactation stage [22], [23], [24]. In addition to these non-genetic effects, there is growing evidence that the individual's genotype may predetermine immunological responses to infection and vaccination [23], [24], [25]. The Ab response to immunization also appears to be often dependent on the adjuvant adopted. Currently, the exact mechanism of action of many adjuvants is still unknown, and research continues to strive to identify the best adjuvant or combination of adjuvants to elicit the correct immune response for a given antigen [26]. Furthermore, selection of carrier proteins used in haptenic vaccines has proven to be greatly important in eliciting potent anti-hapten Ab because it exerts a clear influence on the immune response [27]. It is therefore possible that appropriate vaccine formulations (e.g. different adjuvants or carriers) could stimulate the immunity and surmount deficiencies in low responder cows.

Reduction of AFM_1_ in milk obtained in high responder vaccinated cows was comparable with reductions obtained adding to animal diet the best AFs sequestering agents, which are able to reduce AFM_1_ transfer in the milk up to 50%, depending on the extent of contamination [28], [29]. The results obtained by vaccination, moreover, could be cumulated with the ones eventually achieved by adopting alternative treatments.

Easy detection of anti-AFB_1_ Abs could allow monitoring the immunological status of milk animals in order to determine the protective titre and evaluate the need for booster injections.

Although the schedule of immunization, the nature of adjuvant and carrier, the requirement for boosters and some other factors, such as the fate of AFB_1_ captured by antibodies, should be furtherly investigated, conjugated AnAFB_1_ may represent a possible solution to a global and serious health problem.

## Materials and Methods

### Ethics statement

The research protocol and animal care were in accordance with the EC Council Directive guidelines for animals used for experimental and other scientific purposes [30].

The study has been approved by the local health autority “Azienda Unità Sanitaria Locale di Piacenza” (protocol number 24567) and by the National Ministry of Health according to legislative decree 116/92.

### Preparation of AnAFB_1_

AFB_1_ (Sigma-Aldrich, Inc., St. Louis, MO, USA) was converted to AFB_1_-1(O-carboxymethyl) oxime using a method previously described [15]. Briefly, carboxymethylhydroxylamine•HCl (10 mg, 0.046 mmol) was added to a solution of AFB_1_ (10 mg, 0.032 mmol) in methanol/water/pyridine (4∶1∶1) and the mixture was refluxed at 60°C for 3 h. After maintenance overnight at room temperature, the solution was concentrated under vacuum. The product was purified by flash chromatography (CHCl_3_∶MeOH = 7∶3), and confirmed to be AFB_1_-1(O-carboxymethyl) oxime by UPLC/MS (Acquity, Waters, Milford, MA, USA). The UPLC separation was performed on a reversed-phase C-18 column (UPLC BEH Aquity, Waters; 17 µm×21 mm), eluted at a flow rate of 300 µl/min. Mobile phase consisted of 0.2% formic acid in H_2_O (solvent A) and 0.2% formic acid in acetonitrile (solvent B).

### Cytotoxicity assay of AnAFB_1_ on HepG2

Human hepatoblastoma (HepG2) cell line (BS-TCL-79, ATCC, Rockville, MD, USA) was maintained in tissue culture flasks with Dulbecco's modified Eagle's medium (DMEM) (Sigma-Aldrich) containing 10% fetal calf serum, 1% antibiotics (10,000 U/ml penicillin, 10,000 U/ml streptomycin sulfate), 1% glutamine, and 1 mM sodium pyruvate (Life Technologies, Gaithersburg, MD, USA). Cells were cultured in a humidified incubator under 5% CO_2_ at 37°C and split twice a week. Cell viability was assessed by spectrophotometrically measuring alamarBlue (AB) (Biosource International Inc., Camarillo, CA, USA) reduction by mitochondrial enzyme activity [31]. The day before treatment HepG2 were trypsinized and seeded at a density of 5×10^4^ cells/well in flat bottom 96-wells plates (Costar, Corning Inc., Corning, NY, USA). After 24 h incubation under 5% CO_2_ at 37°C, the medium in each well was discarded and replaced by fresh medium containing increasing dilutions of AnAFB_1_ or AFB_1_, obtained from stock solutions prepared in dimethylsulfoxide (DMSO). All experiments included both untreated and solvent control cultures. After 24 h of treatment, the medium was discarded and cells were washed twice with PBS. Cells were then incubated for 2 h at 37°C under 5% CO_2_ in 100 µl of incubation medium along with 10% (v/v) AB. Absorbance values were measured at 570/595 nm with a Sunrise Absorbance Reader (Tecan Italia Srl., Milan, Italy). The values were corrected for background of negative controls containing medium without cells. Treated cell viability was calculated in comparison to untreated cultures, assumed to be 100%.

### In vitro mutagenicity assay of AnAFB_1_ in *Salmonella typhimurium*

Mutagenicity of AnAFB_1_, in comparison with AFB_1_, was assessed in the reverse mutation assay by the standard plate incorporation method developed by Maron and Ames [32]. The compounds were tested on His^−^ *S. typhimurium* TA 98 and TA 100 (kindly donated by Dott. Cassoni, ARPA, Parma, Italy) with *in vitro* extracellular microsomal activation (S9 from Aroclor 1254-induced rats, Moltox Inc., Boone, NC, USA). Overnight cultures of the test strains were grown for 8–16 h in Nutrient Broth n.2 (Fluka Chemika, Buchs, Switzerland) to a cell density of approximately 10^9^ CFU/ml. Three concentrations (100, 200 and 500 ng/plate) for AnAFB_1_ and two concentrations (100 and 200 ng/plate) for AFB_1_ were tested, with three replicate plates at each dose. The compounds were dissolved in DMSO and diluted in distilled water immediately prior to testing. 0.1 ml each of the test sample and bacterial suspension with 0.5 ml PBS (pH 7.4) or 0.5 ml of the metabolic system S9 were added to 2 ml of molten top agar (45°C) supplemented with 0.05 mM L-histidine and 0.05 mM d-biotin and subsequently poured onto minimal glucose agar plates (15 ml/plate). After 48 h at 37°C the colonies (His^+^ revertants) in each plate were counted. Solvent alone served as negative control (spontaneous mutation frequency) while AFB_1_ was included as a positive control [33]. A mutagenic potential was assumed, if a two-fold or greater increase was seen in the number of revertant colonies of the treated cultures in comparison to negative controls.

### Conjugation of AnAFB_1_ to KLH

AnAFB_1_ was conjugated to KLH (Sigma-Aldrich) to be used as immunogen. The coupling reaction was carried out using a method previously described [34]. N-hydroxysuccinimide (1.1 mg) and N,N′-diisopropylcarbodiimide (1.45 µl), dissolved in 600 µl of dimethylformamide (DMF), were added to AnAFB_1_ (2 mg) in 600 µl of dry dichloromethane at 0°C, followed by 4-(dimethylamino)pyridine (1 mg). Then, the active ester was slowly added to a pre-cooled aqueous buffered solution (31 mM Na_2_HPO_4_, pH 9.1) containing 20 mg KLH and not more than 10% (v/v) DMF. The mixture was kept at 4°C overnight, and then the conjugate was separated from unreacted reagents and byproducts, desalted and extensively dialyzed against PBS by using a 10 kDa cut-off centrifugal filter tube (Microcon YM-10, Millipore Corporation, Bedford, MA, USA). The protein concentration was determined by using the Bradford method, using BSA (Sigma-Aldrich) as external standard. In order to estimate the AFB_1_ loading on KLH, UV absorbance was measured at 363 nm, assuming that the absorbance of the conjugated AFB_1_ was the same as the unmodified toxin (ε = 21,800 L·mol^−1^·cm^−1^). The ratio between the concentration of the bound toxin and that of the protein gave the loading degree of the conjugate.

### Animal immunization

Two groups of six multiparous lactating Holstein Friesian dairy cows were used in the present study. Cows were housed in a free stall barn (CERZOO research and experimental centre, San Bonico, Italy) and had free access to water. The diet was formulated according to the nutrient requirements of dairy cattle for an average cow weighting 650 kg, 140 days in milk (DIM) and a 35 kg milk yield (3.8% fat and 3.35% protein) [35]. The diet, composed both by forages and concentrate (total mixed ration), was fed ad libitum. Cows were milked twice a day and individual milk yield was recorded at every milking (Afimilk system, S.A.E. Afikim, Kibbutz Afikim, Israel). The animals were regularly inspected by the local competent authority; official intradermal tuberculin test was carried out and interpreted according to EC Commission Regulations [36]. Each cow was immunized by intramuscular (*i.m.*) neck injection with an 1 ml emulsion of 500 µg of either AnAFB_1_-KLH (vaccinated animals) or KLH (control animals) in complete Freund's adjuvant (Sigma-Aldrich) in a 1∶1 (v/v) ratio. Priming injection was done at 85±21 DIM and was followed by three similar dose booster injections in incomplete Freund's adjuvant at three week intervals. Animals were bled via the jugular artery prior to immunization and weekly thereafter. The blood was allowed to clot 60 min at 37°C, and the serum was obtained by centrifugation (1500 *g*, 10 min) and stored at −20°C until assay.

### Enzyme-linked immunosorbent assay (ELISA)

For titration of specific antibody, wells of polystyrene microtiter plates (Costar) were coated overnight (4°C) with 50 µl of AFB_1_-BSA conjugate (Sigma-Aldrich) or BSA control protein, 20 µg/ml, in 0.05 M sodium carbonate-bicarbonate buffer (pH 9.6). Plates were washed at this point and after each incubation step with 100 µl of PBS containing 0.05% Tween 20. Wells were blocked for 60 min at 37°C with 100 µl of 1% (w/v) gelatin from porcine skin (Type A; Sigma-Aldrich) in PBS. To each well, 50 µl of serially diluted immune serum or control (pre-immune serum) was added, gently mixed, and incubated at 37°C for 90 min. 50 µl of rabbit anti-bovine IgG (whole molecule) peroxidase conjugate Abs (Sigma-Aldrich, product number A7414) diluted 1∶25,000 in PBS were added to each well. After 60 min incubation at 37°C, 50 µl of freshly prepared cromogen/substrate solution, constituted by 1 mg of 3,3′, 5,5′-tetramethylbenzidine dihydrochloride and 2 µl of 30% H_2_O_2_ in 10 ml of citric buffer (51 mM Na_2_HPO_4_, 24 mM citric acid, pH 5.0), were added. The reaction was stopped after ten minutes with 25 µl of 0.5 M H_2_SO_4_. The optical density (OD) at 450 nm was read by using a Multiskan Ascent (Labsystems, Helsinki, Finland) and the titer of each immune serum was defined as the inverse of the highest dilution that gave 0.1 OD above the pre-immune serum at the same dilution. To compensate for between-plate variability, individual plates were normalized to the mean of the appropriate positive control.

A competitive indirect ELISA (ci-ELISA) was used to assess the cross-reactivity of anti-AFB_1_ Abs in cow's sera with other AFs. Microtiter plates were coated with AFB_1_-BSA and blocked as described above. Increasing concentrations of AFB_1_, AFB_2_, AFG_1_, AFG_2_, or AFM_1_ in 25 µl PBS were mixed with a 25 µl volume of immune serum (diluted 1∶400 in PBS) in the wells of the microtiter plates and incubated for 1 h at 37°C. Bound Abs were determined by the addition of anti-bovine IgG peroxidase conjugate as described above. The absorbance in the assay without free AFs was assumed as the maximal value. AF concentration causing 50% inhibition (IC_50_) of binding of the immune serum to AFB_1_-BSA was calculated by variable slope nonlinear regression analysis of curves obtained by plotting the percent absorbance values versus log AF concentration using GraphPhad Prism 4.01 software (San Diego, CA, USA). The relative cross-reactivity of the immune serum with different AFs was calculated as (IC_50_ of AFB_1_/IC_50_ of other AF)×100.

### Quantification of AFB_1_ in feeds

Ten grams of dried feed was suspended in 100 ml acetone∶water solution (85∶15), shaken at 150 r.p.m. for 45 min (Universal table Shaker 709, ASAL srl, Milan, Italy), filtered (Schleicher & Schuell 595 ½ filter paper, Dassel, Germany), and 5 ml was loaded on an immunoaffinity column (Aflatoxin Easy-extract, Rhone Diagnostics Technologies, Glasgow, UK) and the column washed with 45 ml bidistilled water. The column was further washed with 5 ml water and bound AFB_1_ eluted with 2.5 ml of methanol. The extract was dried under nitrogen, redissolved in 1 ml acetonitrile∶water (25∶75) solution and filtered (Millipore Corporation; HV 0.45 µm). HPLC analysis was performed with a Perkin Elmer LC (Perkin Elmer, Norwalk, CT, USA) equipped with an LC-200 pump and a Jasco FP-1520 fluorescence detector (Jasco, Tokyo, Japan) set at 365 nm (excitation) and 440 nm (emission). Calibration was performed as a function of standard AFB_1_ (Sigma-Aldrich) concentrations. Standard solutions were prepared and checked according to the AOAC method 970.44 [37]. AFB_1_ was separated with a reverse-phase C18 Superspher column (Merck, Darmstadt, Germany; 4 µm particle size, 125×4 mm i.d.) at room temperature, with water∶acetonitrile∶methanol (59∶15∶26) mobile phase at a flow rate of 1 ml/min.

### Treatment of dairy cows with AFB_1_

The study consisted of two successive intoxication periods: the first, starting 1 week after the last booster injection, carried out at mid lactation stage (155±21 DIM, average milk yield of 32.1±5.6 kg/day *per* cow), and the second at late lactation stage (246±21 DIM, average milk production of 28.8±3.1 kg/day *per* cow).

The basal diet had an AFB_1_ content of 0.50 µg kg^−1^ in the first and 0.55 µg kg^−1^ in the second experimental period, corresponding to about 11.5–12.7 µg *per* cow *per* day based on an average ingestion of 23 kg dry matter *per* cow *per* day. Corn meal naturally hypercontaminated was diluted in about 300 g of AFB_1_-free soy bean meal to obtain a bolus, which gave a calculated daily AFB_1_ total ingestion of 144 µg and 145 µg, respectively.

Experimental periods lasted 16 days, consisting of 11 days of intoxication and 5 days of clearance (no AFB_1_ in the diet). Individual milk samples were collected at day 0, 1, 3, 5, 7, 9, 11, 12, 14, and 16. A representative sample for each day of milking was then obtained and stored at −18°C for subsequent analysis. Basal diet samples were collected on days 0 and 11 of each experimental period, dried at 55°C in a ventilated oven to constant weight, and then ground with a 1 mm sieve (Thomas-Wiley Laboratory Mill, Arthur H. Thomas Co., Philadelphia, PA, USA) and frozen until analysed for AFs.

### Quantification of AFM_1_ in milk samples

Milk samples (50 ml) were defatted by centrifugation (7,000 r.p.m. for 10 min at 4°C) and filtered with Schleicher & Schuell 595 ½ filter paper (Dassel). Then, 20 ml was passed through the immunoaffinity column (Aflatoxin Easy-extract) and AFM_1_ was quantified by HPLC, following separation with a reverse-phase C18 LiChrosper 100 column (Merck, Darmstadt, Germany; 5 µm particle size, 125×4 mm i.d.) at room temperature, with water∶acetonitrile (75∶25) mobile phase at a flow rate of 1 ml/min. AFM_1_ (Sigma-Aldrich) was used as calibration standard, as previously described for AFB_1_ quantification.

### Statistical analyses

All data are presented as means ± standard deviations (SD). Differences between immunization groups and the results of the cytotoxicity and mutagenicity assays were analysed using the Student's *t* test. Concerning the excretion pattern of AFM_1_ into milk, the steady-state condition was determined in agreement to Littell et al. [38]. AFM_1_ milk concentration at the plateau condition was subjected to Analysis of Variance (ANOVA). The statistical model included fixed effects of treatment, time of measurement and the treatment×time of measurement interactions, with cow as the random variable. Differences between means were accepted as significant if p<0.05.
